# Cutaneous Atrophy Following Corticosteroid Injections for Tendonitis: Report of Two Cases

**DOI:** 10.2196/67921

**Published:** 2025-02-13

**Authors:** Rebecca Colwell, Mitchell Gullickson, Jonathan Cutlan, Erik Stratman

**Affiliations:** 1 Marshfield Clinic Health System, 1000 N Oak Ave, Marshfield, WI, 54449, United States, 1 715-387-5311; 2 School of Medicine and Health Sciences, University of North Dakota, Grand Forks, ND, United States

**Keywords:** lipoatrophy, cutaneous atrophy, corticosteroid, adverse effects, tendonitis, musculoskeletal

## Abstract

Cutaneous atrophy resulting from corticosteroid injections for musculoskeletal indications is an underrecognized adverse effect among orthopedists and dermatologists. We present two cases of cutaneous atrophy following corticosteroid injections for wrist tendonitis. Patients presenting with cutaneous atrophy following orthopedic corticosteroid injections may be misdiagnosed with linear morphea, atrophoderma, or vascular disorders and receive unnecessary workups and delays in appropriate management. Dermatologists play an essential role in the evaluation of these patients.

## Introduction

Injectable corticosteroids are commonly used to treat musculoskeletal conditions, including tendonitis [1]. Common adverse reactions to corticosteroid injections include atrophy, depigmentation, and cellulitis [1]. Skin depigmentation is a well-recognized adverse effect of corticosteroid injections, but atrophy is underrecognized. Atrophy typically manifests 2-4 months following the injection but may be delayed up to a year [2]. The pathophysiology of soft tissue atrophy and hypopigmentation is hypothesized to stem from macrophage-induced breakdown of adipose tissue, impaired function of melanocytes, and decreased synthesis of type I and type III collagen [3].

Dermatologists are familiar with the risks of cutaneous atrophy due to topical, intralesional, and intramuscular corticosteroid use from dermatologist-initiated treatments but may be less familiar with adverse effects associated with orthopedic uses. We present two patients with pronounced cutaneous atrophy of the injected wrists after corticosteroid injections for tendonitis.

## Case 1

**Figure 1.**
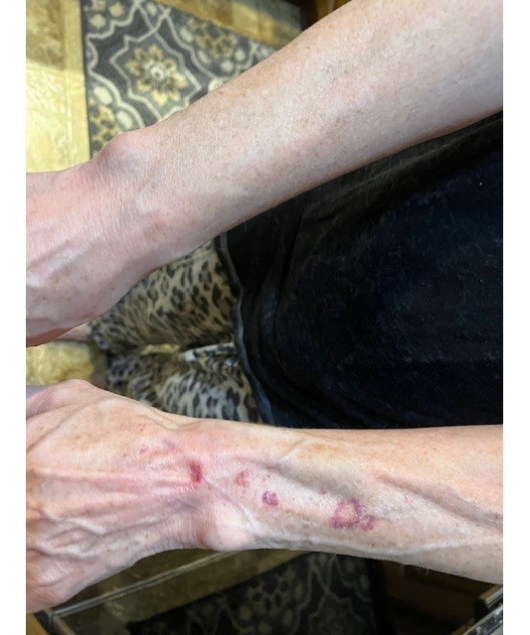
The left forearm shows prominent subcutaneous atrophy and purpura.

A 58-year-old woman presented to the Department of Dermatology for evaluation of skin fragility and discoloration over her left extensor forearm. Six months earlier, she received a 1-mL injection of a suspension of 0.5 mL of 40 mg/mL triamcinolone mixed with 0.5 mL of 1% lidocaine in the extensor carpi radialis brevis and extensor carpi radialis longus to treat wrist tendonitis. This initially relieved her pain, but 2 months following the injection, she noticed skin discoloration and soft tissue atrophy at her left distal forearm near the injection site. She noted proximal extension of the forearm atrophy. A physical examination revealed linear epidermal, dermal, and subcutaneous tissue atrophy; scattered ecchymoses; and cigarette-paper wrinkling of the skin on the left lateral wrist, forearm, and hand (Figure 1).

**Figure 2.**
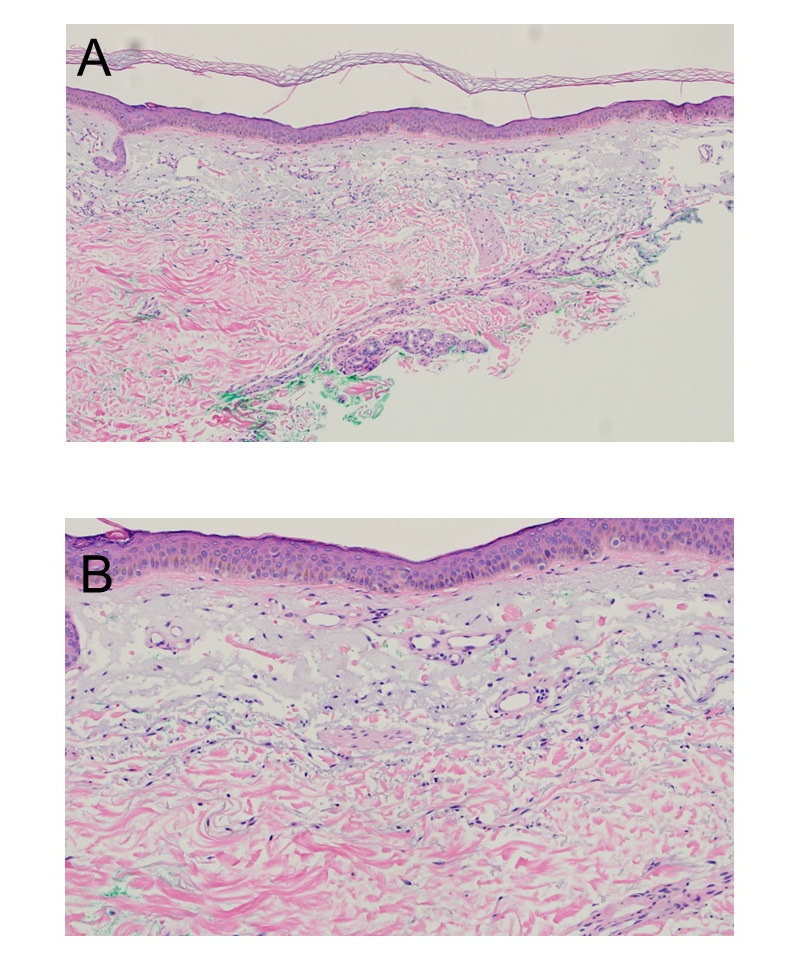
Epidermal atrophy, solar elastosis, and vascular prominence. A, Hematoxylin and eosin stain, original magnification x 100. B, Hematoxylin and eosin stain, original magnification x 200.

Neither the patient nor her care team connected the findings to previous wrist injections because the findings extended several centimeters proximal to the original injection sites. An electromyography test demonstrated no abnormalities. A 3-mm punch biopsy demonstrated mild epidermal atrophy, dermal elastosis, and slight vascular prominence (Figure 2).

**Figure 3.**
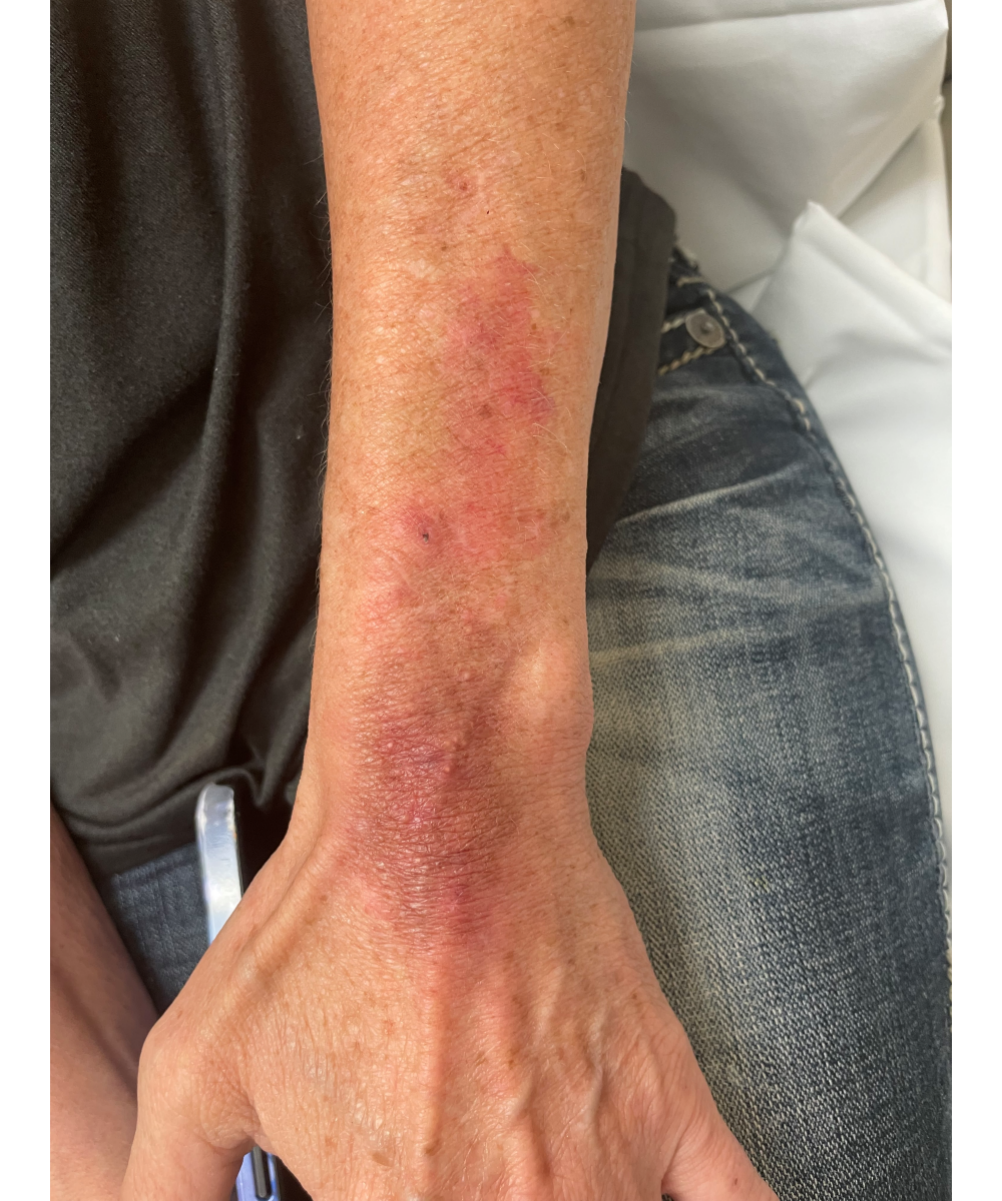
Persistent subcutaneous atrophy, telangiectasias, and hemosiderin deposition over the left forearm.

Active monitoring was chosen for management. One year later, the patient still experienced skin fragility, distal arm lipoatrophy, and wrist weakness. A physical examination showed persistent epidermal, dermal, and subcutaneous tissue atrophy including some telangiectasias, hemosiderin deposition, and cigarette-paper wrinkling of the skin over the left lateral wrist, proximal dorsal hand, and forearm (Figure 3).

The patient consulted with the Department of Plastic Surgery for autologous fat grafting but declined further treatment.

## Case 2

**Figure 4.**
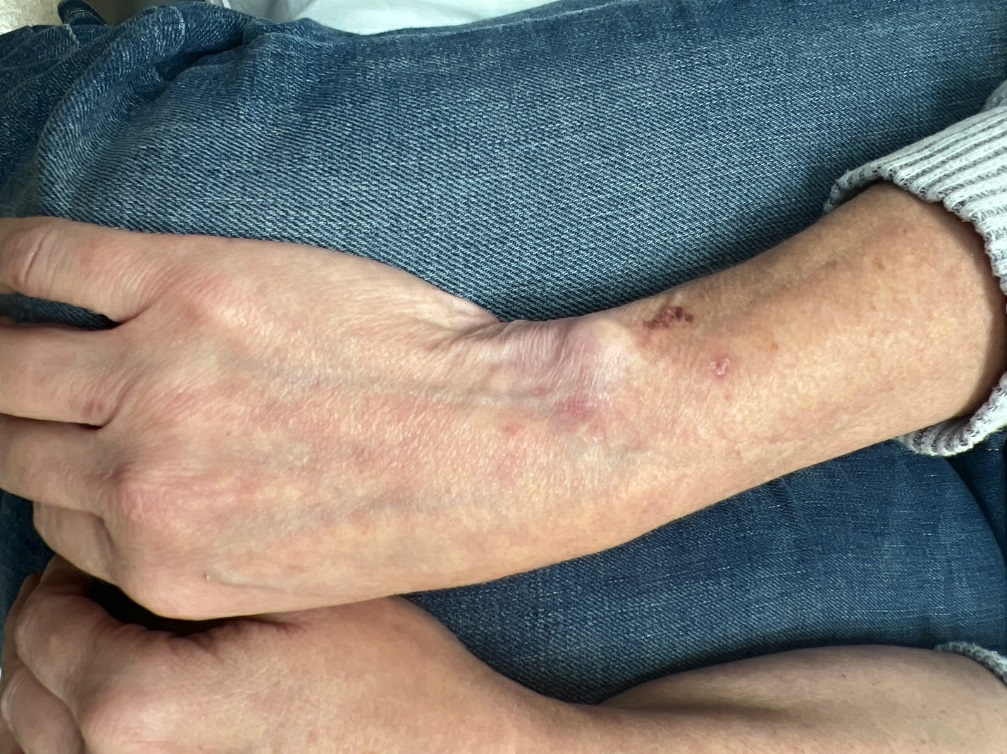
The right wrist shows ulnar prominence secondary to subcutaneous atrophy with overlying purpura.

A 54-year-old woman presented with 3 months of painful, progressive purpura over the forearm skin associated with skin fragility. She received four separate 1-mL injections of a suspension of 0.5 mL of 40 mg/mL triamcinolone mixed with 0.5 mL of 1% lidocaine for extensor carpi ulnaris tendonitis and a partial triangular fibrocartilage complex tear. She noted her distal ulnar head was more prominent and the surrounding skin was hypopigmented. Pain, purpura, and skin fragility began shortly after the fourth injection, prompting a referral to the Department of Dermatology. A physical examination revealed epidermal, dermal, and subcutaneous tissue atrophy with overlying linear purpura (Figure 4).

The patient was recommended to use over-the-counter topical vitamin C and E oils. The patient was offered serial saline injections but declined further treatment at the time of writing this report.

## Ethical Considerations

Both patients provided written consent for their photographs and medical information to be published in print and online, with the understanding that this information may be publicly available.

## Discussion

The timing and location of the patients’ symptoms are most indicative of iatrogenic atrophy after corticosteroid injections. This is an uncommon but known adverse effect of these procedures. Clinicians injecting corticosteroids should advise patients of this risk in their informed consent, particularly when performing superficial injections. The unilateral proximal linear extension of the atrophy and dyspigmentation are often underrecognized as related to the therapeutic injection because the skin atrophy is so extensive and distant from the site of injection. This extension likely occurs secondary to venous or lymphatic diffusion of the insoluble microcrystalline steroid crystals [2]. A glossary of the dermatologic terms described in the report has been provided in Multimedia Appendix 1.

Clinicians can reduce the risks by choosing short-acting more soluble corticosteroids, avoiding injections with unnecessarily high concentrations or volumes of topical steroids, utilizing a 23- to 27-gauge needle to maximize delivery, and considering the use of point-of-care ultrasound—where available—to infiltrate anatomically discreet structures such as tendon sheaths [2].

There are few cases in the literature regarding the efficacy of the therapeutic options for steroid-induced lipoatrophy [2]. Current treatment options include autologous fat grafting, serial saline injections, autologous blood injections, and poly-l-lactic acid injections.

Autologous fat grafting is hypothesized to influence angiogenesis, improving soft tissue quality [4]. However, fat grafting does not address the dyspigmentation, telangiectasia, or epidermal fragility often observed with steroid-induced atrophy. Serial saline injections improve atrophy through the resuspension of steroid crystals, allowing for macrophage-mediated phagocytosis of these crystals [5-7]. Autologous blood injections stimulate cellular and humoral immune response factors such as vascular endothelial growth factor and hepatocyte growth factor [8,9]. Finally, for patients with more limited atrophy, poly-l-lactic acid injections can be administered, with the maximum improvement observed 6 months after injections [10].

Dermatologists managing patients with extensive iatrogenic atrophy cannot overlook the psychosocial impacts this may have. Given the high visibility of the body region affected, significant emotional toll can occur. Functional impairment and persistent weakness may also occur, occasionally with functional impairment and persistent weakness [1]. Medicolegal, workers’ compensation, and risk management conversations are commonly needed, so dermatologists should be prepared for such inquiries and communications.

Extensive cutaneous wrist and forearm skin and soft tissue atrophy may occur in patients undergoing orthopedic wrist injections with corticosteroids. Dermatologists can play a pivotal role in identifying the cause, educating providers who perform these orthopedic procedures, and directing patients to the appropriate treatments.

## Supplementary material

10.2196/67921 Multimedia Appendix 1 Glossary of dermatologic terms.
