# Supplementary material for: Cutaneous Atrophy Following Corticosteroid Injections for Tendonitis: Report of Two Cases
Source: JMIR Dermatol. 2025 Feb 13;8:e67921. doi: 10.2196/67921 (PMC11841998; doi:10.2196/67921)
Supplement: Multimedia Appendix 1 [file derma-v8-e67921-s001.docx]

**Glossary of Dermatologic Terms:**

Linear morphea – an autoimmune disease that causes firm, often sunken, fibrotic changes to the skin.

Atrophoderma – a form of dermal atrophy.

Ecchymoses – bruising or discoloration of the skin caused by bleeding underneath the skin.

Cigarette-paper wrinkling – fine wrinkling of the epidermis due to superficial skin atrophy, giving the appearance of cigarette paper.

Dermal elastosis – degenerative changes of the dermis with increased deposition of disorganized, abnormal elastin.

Telangiectasias – visible, often tortuous, small-caliber (less than 1-mm diameter) blood vessels on the skin surface.

Hemosiderin deposition – brown dyspigmentation of the skin caused by chronic macrophage uptake of iron, usually a byproduct of digested extravasated red blood cells in the skin.
